# Short-Term Effects of Dark Chocolate on Retinal and Choriocapillaris Perfusion in Young, Healthy Subjects Using Optical Coherence Tomography Angiography

**DOI:** 10.3390/nu12030664

**Published:** 2020-02-29

**Authors:** Gianluca Scuderi, Chiara Ciancimino, Fabian D’Apolito, Maurizio Maurizi Enrici, Fabio Guglielmelli, Luca Scuderi, Solmaz Abdolrahimzadeh

**Affiliations:** 1 Ophthalmology Unit, NESMOS Department, St. Andrea Hospital, “Sapienza” University of Rome, Via di Grottarossa 1035/1039, 00189 Rome, Italy; 2 Ophthalmology Unit, Department of Sensory Organs, Policlinico Umberto I, “Sapienza “University of Rome, Viale del Policlinico 155, 00161 Rome, Italy

**Keywords:** dark chocolate, optical coherence tomography angiography, foveal vascular density, whole retinal vascular density, choriocapillaris flow area, flavonoids, superficial retinal plexus, deep retinal plexus

## Abstract

(1) Aim: Contrasting results have been published on the effect of dark chocolate on visual function. The aim of this study was to evaluate retinal and choriocapillaris perfusion, using optical coherence tomography angiography (OCT-A), and visual function in healthy subjects following dark chocolate ingestion. (2) Methods: This prospective randomized study was carried out on 18 healthy young subjects at the St. Andrea Hospital, Sapienza, University of Rome. Visual acuity assessment and a complete ophthalmologic examination were carried out at baseline. In session one, each subject was randomized to eat either a 100 g dark chocolate bar or a 100 g white chocolate bar. In session two, the opposite chocolate was given to each participant. OCT-A and best corrected visual acuity (BCVA) were performed before the chocolate was eaten and repeated 1, 2, and 3 h after that. Retinal vessel density and choriocapillaris flow area were assessed. (3) Results: 18 patients with a mean (SD) age of 26.3 (1.5) years were included. No significant differences between dark or white chocolate were found when evaluating foveal density (%), whole density (%), choriocapillaris flow area, and BCVA. (4) Conclusions: Dark chocolate did not result in significant changes in retinal perfusion and choriocapillaris flow area. However, given the results of other studies showing the positive effects of flavonoids on visual function, further studies are warranted using pure chocolate without other components such as caffeine that can potentially affect results. Furthermore, we cannot rule out the possible benefits of higher doses of flavonoids in dietary supplementation over a more extended period and in a larger patient population.

## 1. Introduction

Polyphenols are organic compounds naturally occurring in plants and constitute a group of more than 8000 molecules with similar chemical structures. Flavonoids are part of the polyphenol family and are generally present in chocolate, cereals, fruits, vegetables, olives, legumes, and in some of the most common beverages such as tea, wine, and beer [1]. 

Various studies have evaluated the association between consumption of polyphenol-rich foods and the incidence of chronic diseases, such as hypertension, dyslipidemias, heart failure, hypercholesterolemia, and diabetes [2,3,4,5,6,7,8,9,10]. Regular consumption of polyphenol-rich food, such as chocolate, vegetables, and fruits, has been linked to a decreased risk of cardiovascular events and strokes [4,8,9]. The role of polyphenols has also been studied in healthy subjects; for example, when taken chronically, chocolate has shown to reduce the oxygen cost of moderately intense cycling [11].

These effects have been associated with the natural antioxidant properties of polyphenols and their impact in improving endothelial function, decreasing coagulation by inhibiting platelet activation and aggregation [12,13,14,15]. The endothelium of blood vessels acts as a barrier, but also synthesizes vasoactive substances, such as nitric oxide (vasodilator) and endothelin-1 (vasoconstrictor). Flavonoids influence the production and metabolism of the endothelial nitric oxide [10,14,15] and have an inhibiting effect on endothelin-1 synthesis, a potent vasoconstrictor [16].

Within the ophthalmology field, different studies have been conducted on polyphenols. Terai et al. [17], in a study on glaucomatous patients, demonstrated a significant improvement in venous vasodilation using dynamic vessel analysis within the control group.

On the effect of polyphenols on visual function, there have been contrasting reports [18,19,20]. Rabin et al. [19], in 2018, reported improved contrast sensitivity within two hours of chocolate consumption, whereas Siedlecki et al. [20] did not find visual acuity or contrast sensitivity improvements at two hours from chocolate ingestion in a similar study in 2019. In this second study, the authors also performed optical coherence tomography angiography (OCT-A) but did not find any significant effect of dark chocolate on retinal perfusion. 

Optical coherence tomography (OCT) has been a groundbreaking imaging method in ophthalmology, as it enables rapid in vivo evaluation of individual retinal layers. It is used routinely in the diagnosis and follow-up of retinal diseases [21,22]. Successive improvement in technology and instrumentation provided the possibility of using enhanced depth imaging (EDI) using spectral domain optical coherence tomography to study the choroid. This method has opened a new window in understanding choroidal physiopathology in normality and disease [23,24,25,26,27,28,29,30]. OCT methods have rapidly improved, and angiography has been included, thus giving rise to OCT-A, which has revolutionized the evaluation of retinal and choroidal perfusion. It is used to evaluate the superficial vascular network, deep vascular network, and choriocapillaris.

The choroid is the principal source of nutrients and oxygen to the retinal pigment epithelium-photoreceptor layer, as the outer retinal layers do not have a vascular network [30,31]. The photoreceptors have a high metabolic activity, and more than 90% of the oxygen received by the retina is used by the photoreceptors. Choroidal circulation provides 90% of the oxygen to the retina during darkness [30]. Blood flow in the choroid is one of the highest per unit weight among the body tissues, approximately ten times higher than the brain [31,32,33,34]; this enables oxygen to be transported across Bruch’s membrane and reach the retinal pigment epithelium. The choriocapillaris forms a thin layer of capillaries in an extensive anastomosed network. Thus, the choroid has a significant role in the tropism of the outer retinal layer and is fundamental in visual function.

Therefore, it may be hypothesized that perfusion changes could be better evidenced in the choroid due to its predominantly vascular nature. Choriocapillaris flow area has not been previously evaluated following chocolate ingestion. Thus, the aim of our study was to determine both retinal and choriocapillaris perfusion alterations following dark chocolate ingestion using optical coherence tomography angiography (OCT-A).

## 2. Materials and Methods

### 2.1. Participants and Study Design

This was a prospective randomized, study on healthy subjects who were enrolled at the St. Andrea Hospital, Sapienza University of Rome. The study was approved by the Institutional Review Board (reference number 5606_2019) and was conducted in accordance with the tenets of the Declaration of Helsinki. All the subjects gave informed consent.

Exclusion criteria were (1) subjects with history of any systemic disease, including diabetes, hypertension, and renal disease, which may affect ocular circulations, (2) subjects with a history of recent or chronic use of medications, including antihistamines or analgesics, (3) subjects with a history of ocular pathology or surgery, (4) subjects with a refractive spherical equivalent above ± 3 diopters, and (5) subjects with intraocular pressure (IOP) above 18 mmHg.

The sample size was calculated at 18 subjects based on Siedlecki et al. [20,35] who determined the change in mean vessel density of approximately 50% in the superficial and 55% in the deep plexus, and an absolute change of 4% in either plexus was set as the cutoff for a significant difference. The resulting sample size for a power of 80% at a 2-sided alpha error of < 0.05 was calculated to be *n* = 18.

**Figure 1:**
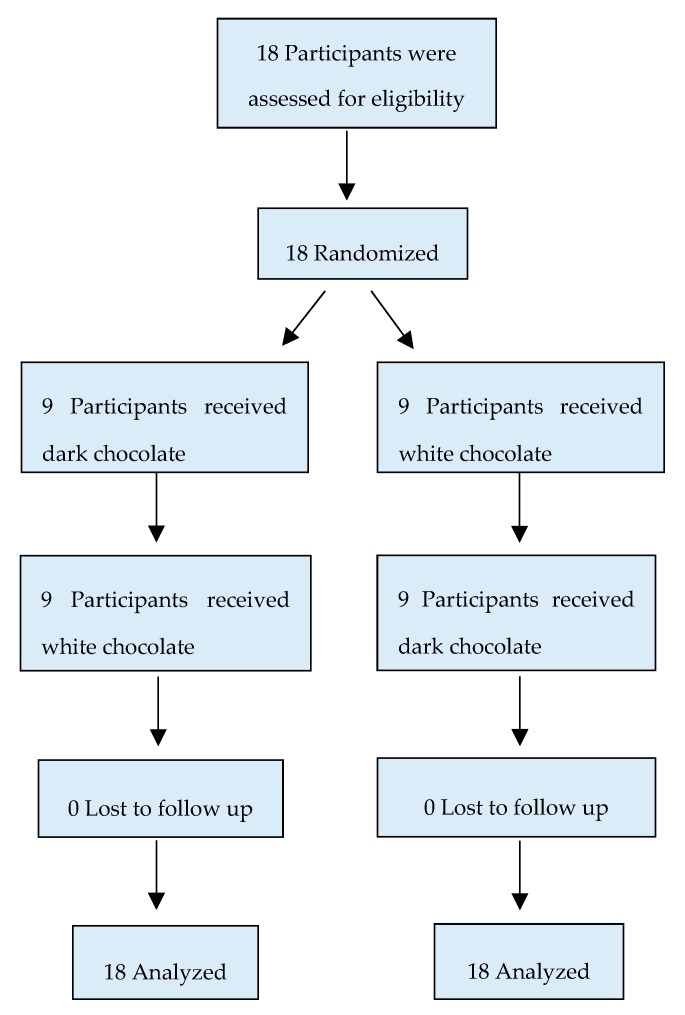
Randomization flow chart of participants in the study.

During the first visit randomization was carried out with forced equal distribution into two groups to determine which type of chocolate patients would start with. On the subsequent visit, the opposite chocolate was given. Figure 1 demonstrates how patients were randomized into two different groups.

### 2.2. Examination

All healthy participants underwent a complete ophthalmologic examination at baseline, before the ingestion of both dark and white chocolate, including BCVA assessment with the Early Treatment Diabetic Retinopathy Study (ETDRS) chart, rebound tonometry [36], slit lamp examination of the anterior segment, fundus examination, and OCT-A imaging.

Each participant was required to participate in two sessions of OCT-A testing, at least 72 h apart. Only one investigator was responsible for randomizing patients and distributing the allocated chocolate. Each patient ingested the chocolate in a separate room and was instructed not to inform the investigators performing the examinations about the type of chocolate they consumed. The subjects were not masked to the type of chocolate, as this was evident on ingestion due to its color and taste. However, the ophthalmologist performing OCT-A and visual acuity assessment was not informed of the type of chocolate consumed by the participants. Blood pressure was not measured before OCT-A examinations as Siedlecki et al. [20] did not find a significant effect of dark chocolate on systemic arterial pressure in their recent report. 

In session one, each subject was randomized to eat either a 100 g dark chocolate bar (Cioccolata Bonajuto, Antica Dolceria Bonajuto, Modica, Italy) with a composition of 447 mg epicatechin, 59 mg catechin, 14 mg quercetin, and 108.8 mg caffeine [37] or a 100 g white chocolate bar (Milka, Kraft food, Mondelēz International, Milan, Italy) that contained only trace quantities of polyphenols (0.04 mg catechin) and caffeine amounts below the level of detection, macronutrients and energy were similar for both types of chocolate [37]. In session two, the other chocolate was given to each participant. OCT-A was performed at 1, 2, and 3 h following chocolate ingestion at every session.

### 2.3. OCT-Angiography

An Optovue RTVue XR Avanti instrument (software version 2017.1.0.151, Optovue Inc., Freemont, CA, USA) based on split spectrum amplitude decorrelation angiography was used to detect macular blood flow. This OCT-Angiography device has a scanning velocity of 70.000 A-scans per second obtaining volumetric 304 × 304 A-scans scans utilizing a light source of 840 nm and an axial resolution of 5 microns. Motion correction technology removes artifacts due to loss of fixation and saccades.

All subjects were requested to fast from midnight before the day of testing, as some foods and beverages, such as caffeine, can affect perfusion. OCT-A imaging was performed between 10:30 to 13:30 to avoid bias due to diurnal variations. Baseline OCT-A was performed on the day of testing before chocolate ingestion.

**Figure 2:**
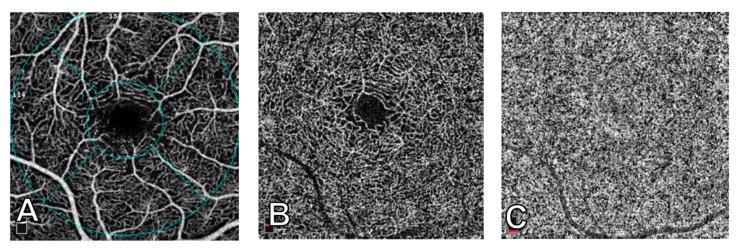
3 mm × 3 mm images of macular perfusion using optical coherence tomography angiography. (**A**) Superficial capillary plexus, showing the early treatment diabetic retinopathy study contour areas: fovea (central circle with a 1 mm radius), temporal, superior, nasal, and inferior. (**B**) Deep capillary plexus. (**C**) Choriocapillaris.

Three-dimensional OCT scans were acquired over a 3 mm × 3 mm macular region. With the use of the DualTrac Motion Correction Technology, which combines real-time tracking, a high-speed infrared camera (30 frames/sec.), and patented post-processing, saccades and minor fixation losses were removed. Scans with low quality were excluded and repeated until good quality was achieved with a minimum cutoff of signal strength ≥ 8/10 [38,39]. Automatic segmentation generated images of the superficial capillary plexus (SCP) and of the deep capillary plexus (DCP). The segmentation boundaries used were from the internal limiting membrane to an outer boundary 9 µm below the inner plexiform layer for the SCP and 9 µm below the inner plexiform layer to 9 µm above the outer plexiform layer for the DCP. Once these scans were obtained, the software calculated vessel density in both SCP and DCP scans as the percentage area occupied by vessels and microvasculature in the selected region. Vessel density was calculated as a whole and then separated into five areas (fovea, temporal, superior, nasal, and inferior) based on the Early Treatment Diabetic Retinopathy Study contour. The parameters used in this study were whole vessel density and foveal vessel density, which represents the central circular area with a 1 mm radius. The choriocapillaris (CC) flow area was calculated automatically as vessel area of CC divided by total area using a 3 mm × 3 mm macular image of the CC layer with the slab automatically segmented at −9 µm to 31 µm offset from Bruch’s membrane [40,41,42]. Figure 2 shows images of the SCP, DCP, and CC.

### 2.4. Statistical Analysis

Statistical analysis was performed with SPSS software package V.21 (SPSS. Inc., Chicago, Illinois, USA). Demographic and clinical characteristics were summarized by standard descriptive summaries (e.g., means and standard deviations for continuous variables, such as age, and percentages for categorical variables, such as gender). The normality of data was determined with the Kolmogorov-Smirnov normality test. If normality was denied, a nonparametric test was used. The primary outcome measure was the change in retinal and choriocapillaris perfusion measured with OCT-A attributable to dark chocolate or white chocolate consumption as measured by OCT-A. The secondary outcome was the change in BCVA as measured with ETDRS charts. The change in DCP foveal density (%), SCP foveal density (%), DCP whole density (%), SCP whole density (%), choriocapillaris flow area, and BCVA 1, 2, and 3 h following the consumption of dark or white chocolate was evaluated with the non-parametric Wilcoxon test for paired data. Results are presented as the mean (standard deviation), as the median interquartile range (IQR), or as the frequency (percentage). A *p* value less than 0.05 was considered statistically significant.

## 3. Results

A total of 18 patients were included. Thus, 36 eyes of 18 patients (8 males and 10 females) with a mean (SD) age of 26.3 (SD 1.5) years (range 24–30 years) were included. Mean BCVA was 61.0 (SD 2.07), mean spherical equivalent was 0.5 (range −2.0–+3.0), mean IOP was 13.83 mmHg (SD 0.88). All 18 patients completed the study and were fully available for analysis. There were no adverse events. The demographic and clinical characteristics of patients are shown in Table 1.

Table 2 shows descriptive statistics for DCP foveal density (%), SCP foveal density (%), DCP whole density (%), SCP whole density (%), choriocapillaris flow area, and BCVA by treatment group at different time points. Data were not normally distributed (Kolmogorov-Smirnov test’s *p* value > 0.05 for all mean differences, data not shown).

Table 3 shows the mean differences in DCP foveal density (%), SCP foveal density (%), DCP whole density (%), SCP whole density (%), choriocapillaris flow area, and BCVA in both groups at different time points. Data were not normally distributed (Kolmogorov-Smirnov test’s *p* value > 0.05 for all mean differences, data not shown).

No significant differences between dark chocolate or white chocolate consumption were found in the DCP foveal density (%), SCP foveal density (%), DCP whole density (%), SCP whole density (%), or choriocapillaris flow area.

No significant differences in BCVA were observed following the ingestion of dark or white chocolate.

## 4. Discussion

In this study, we did not find any significant differences following dark chocolate or white chocolate consumption in the superficial vascular plexus, deep vascular plexus, and choriocapillaris flow area. No significant changes in BCVA were observed. Our results are similar to the results obtained by Siedlecki et al. [20] as regards visual function and retinal perfusion. 

In ophthalmology, consumption of flavonoid-rich cocoa has been associated with visual function and contrast sensitivity enhancement [18,19,20]. The blood concentration of flavonoids is dose-dependent and reaches its highest level at two to three hours from the ingestion of cocoa [18]. Field et al. [18] reported an improvement of cognitive and visual function following consumption of cocoa flavonoids, and recently Rabin et al. [19] reported that dark chocolate improves contrast sensitivity and visual acuity for up to two hours after ingestion. These authors suggested that this could be linked to augmentation of blood flow and increased availability of oxygen. However, these studies are in contrast with the results of Siedlecki et al. [20] and our results, where no improved retinal perfusion using OCT-A was found. An important consideration, that should be taken into account, is the caffeine content present in dark chocolate. Karti et al. [40] found a significantly reduced perfusion in the superficial and the deep retinal plexuses, and also in the choriocapillaris following caffeine consumption. Caffeine could have altered the effect of flavonoids on the retinal and choroidal circulation. In the studies by Taubert et al. [10], Rabin et al. [19], and Siedlecki et al. [20], the possible caffeine content of the products used is neither mentioned nor discussed. This could, in part, explain the conflicting results. The dark chocolate in our study contained 108.5 mg of caffeine, as we used the same product utilized by Grassi et al. [37], who performed high-performance liquid chromatography to analyze chocolate. The amount of caffeine in the dark chocolate bars in our study was about half the dose that Karti et al. [40] used in their study; therefore, it is possible that the vasodilating effect of flavonoids in chocolate might have been limited by the vasoconstrictor effect of caffeine. It is also important to underline that Karti et al. [40] used a 6mm × 6mm macular angiogram of the choriocapillaris layer for analysis, which we did not use due to the poor and un-reliable quality of scans. Furthermore, Karti et al. [40] used a choroidal slab with boundaries 31 to 59 µm below the retinal pigmented epithelium, whereas we used a newer OCT-A software version (2017.1.0.151), which yields a slab at −9 µm to 31 µm offset from Bruch’s membrane, making results difficult to compare.

Another explanation could also be that automated choriocapillaris perfusion only evaluates the choriocapillaris, and not the whole choroidal circulation. Thus, improved technology to reliably evaluate a larger section of the choroid could perhaps provide more significant results and allow a better comparison of our results with other studies.

The rationale of Field et al. [18] as to why no retinal or choroidal perfusion changes were observed in our study or that of Siedlecki et al. [20] is that it may be due to the fact that dark chocolate improves performance on visual and cognitive tasks by increasing cerebral blood flow. This could possibly also explain the results of Rabin et al. [19].

As the choroid is a prevalently vascular structure and had not been previously analyzed with OCT-A following chocolate consumption, we had hypothesized that the choriocapillaris could be an additional, possibly more sensitive, parameter to evaluate changes in perfusion, but we did not find any alterations in the flow area. The caffeine present in the chocolate could have affected this result, especially since the choroid, being a predominantly vascular structure, could be more sensitive to caffeine. 

It has been suggested that introducing a moderate amount of cocoa in a healthy diet can be beneficial not only on the cardiovascular and cerebral level, but also for the ocular system [43]. The optimal amount of daily flavonoids is still undefined; the European Food Safety Authority recommends 200 mg of dark chocolate per day. This can be found in 2.5 g of flavanol-rich cocoa powder, or in 10 g of flavanol-rich dark chocolate [44]. Although we did not find alterations in BCVA or macular perfusion, given the numerous studies showing the positive effects of flavonoids in various conditions, a possible effect of flavonoids on cerebral flow could be involved in the results observed by Field et al. [18] and Rabin et al. [19]. Furthermore, the evaluation of a thicker portion of choroid with customized software in future investigations could provide a more comprehensive evaluation of choroidal flow. The positive effects of flavonoids on macular and choroidal perfusion cannot be completely ruled out, as further studies with caffeine-free chocolate or higher doses of pure flavanol in dietary supplementation are warranted.

## Figures and Tables

**Table 1:** Demographics and clinical characteristics of patients.

Characteristic	Mean (SD)
Age (years)	26.3 (1.53)
IOP (mmHg)	13.9 (0.96)
BCVA	61.0 (2.07)
Sex, N (%)	
Male	10 (56)
Female	8 (44)
**Spherical equivalent**	**N (%)**
+3.00	3 (8.3)
+2.50	2 (5.5)
+2.00	3 (8.3)
+1.50	1 (2.8)
+0.75	1 (2.8)
+0.25	1 (2.8)
No correction	20 (55.5)
−0.25	1 (2.8)
−1.00	1 (2.8)
−1.25	1 (2.8)
−1.50	1 (2.8)
−2.00	1 (2.8)

IOP: intraocular pressure, BCVA: best corrected visual acuity.

**Table 2:** Descriptive statistics at baseline and 1 h, 2 h, and 3 h after dark or white chocolate ingestion.

		White Chocolate		Dark Chocolate	
	Time	Mean (SD)	Median (IQR)	Mean (SD)	Median (IQR)
CC flow area (mm^2^)	Baseline	2.2 (0.1)	2.2 (2.2–2.3)	2.2 (0.1)	2.2 (2.2–2.3)
	1 h	2.2 (0.1)	2.2 (2.2–2.3)	2.2 (0.1)	2.2 (2.2–2.3)
	2 h	2.2 (0.1)	2.2 (2.2–2.2)	2.2 (0.1)	2.2 (2.2–2.3)
	3 h	2.2 (0.1)	2.2 (2.2–2.3)	2.2 (0.1)	2.2 (2.2–2.3)
DCP foveal density (%)	Baseline	40.4 (8.9)	40.7 (35.8–45.1)	40.5 (8.7)	40.7 (34.7–44.9)
	1 h	40.3 (8.6)	40.4 (35.5–45)	40.3 (8.7)	40.9 (34.4–46)
	2 h	39.9 (8.3)	39.9 (35.1–45)	40.3 (8.6)	40.5 (34.1–44.8)
	3 h	40 (8.5)	40.8 (34.8–44.5)	40.3 (8.3)	40.7 (35.6–44.5)
SCP foveal density (%)	Baseline	24 (8.0)	25.1 (18.1–27.6)	23.8 (8.3)	25.1 (17.8–27.9)
	1 h	23.42 (8.0)	23.4 (18.3–27.4)	23.95 (8.3)	24.1 (17.5–28.4)
	2 h	23.28 (7.8)	23 (17.1–27.3)	23.6 (8.0)	23.5 (16.8–28.9)
	3 h	23.20 (8)	24.2 (16.2–27.4)	24.44 (9.5)	23.3 (17.3–28.1)
DCP whole density (%)	Baseline	54.12 (2.82)	54.6 (53.5–55.5)	54.01 (2.21)	54.4 (52.2–55.5)
	1 h	53.53 (2.39)	53.4 (52.7–54.9)	53.57 (2.44)	54.1 (52.4–55.4)
	2 h	53.04 (3.44)	53.8 (51.4–55)	52.94 (2.35)	53.4 (51.1–54.3)
	3 h	53.19 (3.27)	53.7 (52.1–55.2)	52.78 (2.82)	53.2 (51–54.7)
SCP whole density (%)	Baseline	48.9 (2.2)	49.4 (47.6–50.3)	49.0 (2.1)	49.2 (47.9–50.6)
	1 h	48.6 (2.3)	48.7 (47.2–50.3)	48.7 (2.6)	48.3 (46.3–50.5)
	2 h	48.3 (3.1)	48.8 (47.2–50.2)	48.3 (2.3)	48.1 (47.2–50.3)
	3 h	48.3 (3.0)	48.7 (47.4–50.2)	48.0 (2.6)	48.8 (46.5–50)
BCVA (EDTRS)	Baseline	85.3 (0.50)	85 (85–85)	85.1 (0.49)	85 (85–85)
	1 h	85 (0.89)	85 (85–85)	85 (0.81)	85 (84.5–85)
	2 h	85.1 (0.67)	85 (85–85)	85 (0.72)	85 (85–85)
	3 h	85.2 (0.74)	85 (85–85)	85.2 (0.68)	85 (85–85)

CC: choriocapillaris, DCP: deep capillary plexus, SCP: superficial capillary plexus, BCVA: best corrected visual acuity, EDTRS: Early Treatment Diabetic Retinopathy Study.

**Table 3:** Efficacy End Points considered as differences between baseline values and after 1 h, 2 h, and 3 h for dark and white chocolate.

	White Chocolate		Dark Chocolate		*p* Value
	Mean	SD	Mean	SD	
**After 1 h**					
Choriocapillaris flow area (mm^2^)	−0.003	0.064	0.002	0.055	0.736
DCP foveal density (%)	−0.169	1.479	−0.158	1.505	0.818
SCP foveal density (%)	−0.589	2.727	0.147	1.712	0.298
DCP whole density (%)	−0.589	2.359	−0.436	2.726	0.880
SCP whole density (%)	−0.389	1.517	−0.328	2.088	0.915
BCVA (EDTRS)	−0.083	2.091	−0.444	1.930	0.936
**After 2 h**					
Choriocapillaris flow area (mm^2^)	−0.024	0.072	0.006	0.069	0.118
DCP foveal density (%)	−0.592	1.701	−0.122	1.947	0.328
SCP foveal density (%)	−0.725	2.739	−0.203	1.656	0.383
DCP whole density (%)	−1.081	3.450	−1.069	2.587	0.771
SCP whole density (%)	−0.586	2.670	−0.739	1.816	0.317
BCVA (EDTRS)	0.194	2.190	−0.167	1.978	0.834
**After 3 h**					
Choriocapillaris flow area (mm^2^)	0.724	0.078	0.724	0.058	0.724
DCP foveal density (%)	−0.444	2.434	−0.194	1.786	0.371
SCP foveal density (%)	−0.806	2.855	0.642	7.213	0.779
DCP whole density (%)	−0.933	4.144	−1.233	3.224	0.538
SCP whole density (%)	−0.625	2.261	−1.036	1.997	0.583
BCVA (EDTRS)	0.222	2.203	−0.194	2.203	0.941

DCP: deep capillary plexus, SCP: superficial capillary plexus, BCVA: best corrected visual acuity, EDTRS: Early Treatment Diabetic Retinopathy Study.
